# Dual-Armed Oncolytic Myxoma Virus Encoding IFN-γ and CD47 Promotes Lymphocyte Infiltration and Tumor Suppression of Syngeneic Murine Melanoma

**DOI:** 10.3390/cancers15194703

**Published:** 2023-09-24

**Authors:** Jong Kyu Woo, Tae-Geuk Kim, Na Yeon Im, Ka-Yeon Son, Minhyeon Cho, Yeo Jin Jeong, Jeong-Im Hong, BoRim Kang, Gansukh Enkhtaivan, Nam-Hyuk Cho, Tommy Alain, Dong Guk Park, Yeon-Sook Lee

**Affiliations:** 1 ViroCure, #502, Ace TwinTower 1, 285 Digital-ro, Guro-gu, Seoul 08381, Republic of Korea; 2 Department of Microbiology and Immunology, College of Medicine, Seoul National University, Seoul 08826, Republic of Korea; 3 Department of Biochemistry, Microbiology and Immunology, Children’s Hospital of Eastern Ontario Research Institute, University of Ottawa, Ottawa, ON K1N 6N5, Canada; 4 Department of Surgery, Dankook University Hospital, Cheonan 31116, Republic of Korea

**Keywords:** oncolytic virus, Armed-Myxoma virus, CD47, IFN-γ, tumor-infiltrating lymphocytes (TIL), Foxp3+ CD4+ regulatory T-cell (T-reg), melanoma

## Abstract

**Simple Summary:**

This study explores using engineered Myxoma virues (MyxV) encoding CD47 and IFN-γ to fight tumors and boost immune responses in the tumor environment. The findings show that the modified MyxV effectively infects and kills cancer cells, triggers immune responses, and reduces immune-suppressing cells. The dual-armed MyxV increases the presence and activity of T-cells in tumors. When combined with αPD-L1, a known immune therapy, MyxV effectively slows tumor growth and extends survival. These results highlight MyxV’s potential as an oncolytic therapy. Further research to enhance its therapeutic efficacy and to determine anti-tumoral mechanisms, will offer a promising avenue for cancer treatment.

**Abstract:**

Myxoma virus (MyxV) is a rabbit-specific poxvirus. However, its ability to selectively target tumor cells has established it as a safe and effective anticancer therapy. To strengthen its preclinical efficacy, transgenes that can prolong cancer cell infection and enhance anti-tumor effector functions are currently being investigated. We engineered MyxV armed with CD47, to turn on a ‘do not eat me’ signal within infected cells with actively replicating viruses, and with IFN-γ to further activate host immune anticancer responses. Tumor suppressive activities were significantly enhanced by the dual-armed MyxV_CD47/IFN-γ compared to parental MyxV or single-armed MyxV_CD47 or MyxV_IFN-γ. In addition, significant increases in IFN-γ+ CD8+T-cells and CD4+ T-cells populations within tumor-infiltrating lymphocytes (TIL) were observed after MyxV_CD47/IFN-γ treatment. Notably, all groups treated with MyxV showed a marked reduction in Foxp3+ CD4+ regulatory T-cells (Tregs) within TIL. We also show that MyxV infection induces PD-L1 up-regulation in cancer cells, and combinational treatment of MyxV with anti-mouse PD-L1 antibodies (αPD-L1) further controlled tumor burden and increased survival in the syngeneic melanoma model B16F10. Our data demonstrate that a CD47 and IFNγ dual-armed MyxV is an effective oncolytic viral immunotherapeutic. These findings strongly support further preclinical investigations to develop next-generation MyxV-based immunotherapy approaches.

## 1. Introduction

The host immune system is a core defense against cancer development and progression, and immune-based therapeutics demonstrate high promises as cancer treatments. Recent cancer immunotherapies targeting immune checkpoint inhibitors (ICIs) such as anti-programmed cell death-1 (PD-1) and anti-programmed cell death ligand-1 (PD-L1) antibodies have led to durable remission across a wide variety of different cancer types. However, various tumors are immunogenically “cold”, exhibiting limited mutational load and neoantigen expression [1,2]. In that regard, most solid tumors, and notably late-stage or metastases, are not sufficiently sensitive to ICI-mediated immune therapy due to poor infiltration of cytotoxic T-cells and a high immunosuppressive tumor microenvironment (TME) [3,4]. Therefore, approaches to alter the immunologically suppressed TME are actively investigated.

Oncolytic virus (OV) therapy utilizes wild-type or genetically modified viruses that are selectively replicating in tumor cells, and that drive an immunotherapeutic response amenable to the treatment of various cancers [5]. OVs exert their therapeutic effects through direct cytolysis, resulting in the release of tumor-specific antigens within the TME. This attracts an immunogenic milieu that promotes systemic immune responses against released tumor antigens [6,7,8]. Several oncolytic viruses have been evaluated in preclinical or clinical studies for treating cancers such as melanoma, head and neck, lung, cervical, and glioma [9,10,11]. Despite current OV therapies being promising approaches to render tumors “hot”, additional strategies and viral backbones should be developed and assessed.

Myxoma virus (MyxV) belongs to the *Leperipoxvius* genus and is a rabbit-specific poxvirus studied for its oncolytic potential [9,12]. MyxV is an excellent platform for generating genetically modified viruses for therapeutic, immune-modulating, gene expression because of its large and stable double-stranded DNA genome. For instance, a recent MyxV bioengineered to express TNF and TNFSF14 demonstrated strong responses as a stand-alone cancer therapy and when combined with immunotherapy in several cancer models [13,14,15]. We hypothesized that prolonged MyxV replication within tumor tissues could further support the critical expression of therapeutic transgenes, and the development of efficient anticancer immunity [16]. In this regard, we constructed a dual-armed MyxV encoding the murine Integrin-associated protein (mCD47) and the murine Interferon-γ (mIFN-γ) (MyxV_CD47/IFN-γ). The aim was to take advantage of the functions of overexpressed CD47 to limit the rapid elimination of MyxV-infected cells, and IFN-γ to fortify the anticancer immunity induced by this OV. CD47 is a transmembrane protein that sends a “do not eat me” signal to phagocytes to evade phagocytosis and thus prevents the prompt removal of infected cells [17,18,19,20]. IFNs are a large family of cytokines and have been discovered more than 20 members in mammals. They are grouped into three types, I, II, and III, determined based on their cellular receptors. The interferon-γ (IFN-γ) is the only type II IFN produced by immune cells such as natural killer (NK) cells, T-cells, and macrophages. It is a potent immune regulator against cancer cells that induces adaptive immune response through increased antigen presentation and T-cell activation [21]. Many studies showed that expression of IFN-γ develops the protective immune response against cancers through improved immune surveillance [22,23]. An IFN-γ-armed MyxV is a strategy to effectively deliver IFN-γ, which has a short half-life and a difficulty to be accumulated at the tumor site [24,25]. We proposed that delay of clearance of MyxV replicating cells by CD47 signal could allow for the accumulation of IFN-γ and lead to better clinical outcomes.

Here we report the anticancer efficacy of an oncolytic MyxV simultaneously expressing CD47, to avoid an antiviral immune response, and IFN-γ, to boost effective anti-tumoral immunity as a stand-alone treatment, or with αPD-L1 combination therapy in melanoma models. With the use of novel immunotherapies and molecular targeted therapies, the 5-year overall survival rate of advanced or metastatic melanoma has increased to greater than 50%. However, there remains a critical unmet need for new therapies that function via alternative immune activation mechanisms with better safety. The efficacy of MyxV_CD47/IFN-γ in B16F10 mouse melanoma syngeneic mouse models resulted in strong tumor regression and prolonged mouse survival. Importantly, analyses of tumor-infiltrating lymphocytes (TILs) revealed that this novel OV favorably altered the immune profile of the TME. The data propose to develop the dual-armed MyxV_CD47/IFN-γ as an efficient therapeutic agent for solid tumors.

## 2. Materials and Methods

### 2.1. Cell Lines

The murine cancer cell lines B16F10, MC38, CT26, and 4T1 were obtained from the Korea cell line bank and cultured in DMEM (Welgene, Gyeongsan, Republic of Korea) supplemented with 10% FBS. The rabbit cell line RK13 was obtained from ATCC and maintained in DMEM supplemented with 10% FBS (Gibco, Gibco, Paisley, Scotland, UK).

### 2.2. Development of Armed MyxV

Parental MyxV_0100 was generated by inserting the poxvirus synthetic early/late (sE/L) promoter and mCherry expression cassette inserted in the m135 and m136 intergenic locus in a wild-type virus (Lausanne strain), which was purchased from ATCC. The mCherry serves as a marker for MyxV infection and replication in cells. The single Armed MyxV_CD47 or MyxV_IFN-γ were generated by inserting the respective gene expression cassette under the control of a sE/L promoter. MyxV_CD47/IFN-γ construction was designed as CD47, and the IFN-γ gene was linked by a 2A “self-cleaving” peptide sequence, and expression was controlled by the sE/L promoter. A gene expression cassette was inserted immediately downstream of the mCherry expression cassette. In brief, the RK13 cells were seeded the day before and infected with 0.01 MOI of MyxV_0100. After 2–3 h of the virus infection, the Ribonucleoprotein (RNP) complex of Cas9 (Toolgen, Seoul, Republic of Korea), guide RNA (5′-GCCAGACTCCGGAACTATGAAGG-3′, 5′-ATACAGACTCCGACGTACGAAGG-3′) (Toolgen), and the PCR-amplified donor fragments was transfected into RK13 cells. The recombinant viruses were isolated by picking red fluorescent positive MyxV-induced foci after 2–5 days. The collected foci were frozen and thawed thrice and infected again into freshly cultured RK cells in 6 well plates. Final isolation of the recombinant virus was observed after 4–6 rounds of picking.

### 2.3. Virus Production and TCID50

The parental MyxV_0100 or armed MyxVs were produced in RK13 cells. MyxV production and titration methods were previously reported [14,26]. Briefly, RK13 cells were infected at 0.2 MOI (TCID50). When cytopathic effects were visible, cells were harvested and subjected to three cycles of freeze-thaw and sonicated. Cell debris were removed by centrifugation. The virus-contained homogenates were subjected to ultracentrifugation using a 40% sucrose cushion, and collected pellets were layered onto the 28, 32, 36, 40, and 46% sucrose gradient cushion, ultracentrifuged, and carefully collected virus bands between 36 and 40% sucrose. The quantity of infectious viral particles was determined by titration on RK13 cells. Serial dilution of MyxV infected RK13 cells, and six days later, fluorescent foci were counted using an inverted fluorescence microscope.

### 2.4. Animal Experiment

The animal experiment schedule of this study was carried out with similar to the previous studies [6,27]. The Ethics Committee of Virocure approved all animal experiment protocols (IACUC No. VRC-IACUC-2208) and conducted according to the National Research Council Guide for the Care and Use of Laboratory Animals, Eight Edition. 2011. Six weeks-old immunocompetent female C57Bl6/J mice were purchased from Orient Bio (Seongnam, Republic of Korea). The C57BL6 mice were subcutaneously inoculated with 3 × 10^5^/100 μL B16F10 murine melanoma cells in the right flank. The cells were mixed with Matrigel (BD Bioscience, San Jose, CA, USA) at a 1:1 ratio. Once palpable tumors reached 100 mm^3^, mice were randomly divided and received PBS, 10^7^ (TCID50) of MyxV_0100, 10^7^ (TCID50) of MyxV_CD47, 10^7^ (TCID50) of MyxV_IFN-γ, and 10^7^ (TCID50) of MyxV_CD47/IFN-γ via intratumoral of in a volume of 50µL at days 1, 3, and 5. In combination treatment, from the second MyxV administration, 10 mg/kg of α-PDL1 (BioXcell, Lebanon, NH, USA) antibody was administered intraperitoneally, with a total of three times at two-day intervals. Tumor volumes and body weights were recorded every three days. For analysis of TIL, three animals from each group were sacrificed on day 9 for FACS analysis. For survival analyses, five to ten mice were used, and mice were monitored for 33 days.

### 2.5. qRT-PCR

RNA isolation was conducted from MyxV-infected murine cancer cell lines using TrI-zol™ (Invitrogen, Waltham, MA, USA). Obtained RNA was subjected to cDNA synthesis using PrimeScript™ 1st strand cDNA Synthesis Kit (Takara, Shiga, Japan). qRT-PCR was performed using the SensiFAST SYBR Lo-Rox Kit (Bioline, London, UK) according to the manufacturer’s protocol. The primer sequences used in the experiment are shown in Supplementary Table S1.

### 2.6. IFN-γ ELISA

The conditioned medium was collected from MyxV-infected murine cancer cells and stored at −80 °C until use. Mouse IFN-γ in the conditioned media was quantified using the ELISA MAX™ Deluxe Set Mouse IFN-γ (Biolegend, San Diego, CA, USA) according to the manufacturer’s protocol.

### 2.7. IHC and Immunofluorescence Staining

Tumor tissues were fixed with 4% (*w*/*v*) PFA (Biosesang, Seongnam, Republic of Korea) and embedded in paraffin blocks. Sections were cut at 5 μm from paraffin-embedded blocks. For immunohistochemistry, the rehydrated sections were boiled in citrate buffer (10 mM citric acid buffer, pH 6.0) for antigen retrieval, and sections were blocked w/Antibody Diluent (ThermoFisher, Waltham, MA, USA). The pretreated sections were incubated with primary antibodies at 4C overnight, and antibody binding targets were visualized by the 2-step plus Poly-HRP Anti Mouse/Rabbit IgG Detection System (Elabscience, Houston, TX, USA) according to the manufacturer’s protocol. All images were obtained through the PANNORAMIC 250 Flash III automatic digital slide scanner (3DHISTECH, Budapest, Hungary). For immunofluorescence staining, the pretreated sections or cell-coated coverslips were incubated with fluorochrome-labeled primary antibodies at 4 °C overnight. The stained slides were mounted with Fluoroshield™ with DAPI mounting medium (ImmunoBioScience, Mukitteo, WA, USA). The image was captured on Zeiss LSM confocal microscope (Carl Zeiss, Jena, Germany). A list of the antibodies used can be found in Supplementary Table S2.

### 2.8. TIL Isolation and FACS Analysis

Mouse tumors were collected, mechanically dissected with surgical scissors, and digested with dissociation buffers (10% FBS in DMEM supplemented with 0.1 mg/mL of hyaluronidase (Sigma-Aldrich, St. Louis, MO, USA) 1 mg/mL of collagenase IV (Worthington-Biochem, Lakewood, NJ, USA), and 300 unit/mL of DNase I (Worthington-Biochem)) for one hour at 37 °C. The resulting tumor single-cell suspensions were immunostained to analyze the different cell populations. Single suspended tumor cells were incubated in Fc receptor binding inhibitor (Invitrogen, Carlsbad, CA, USA) for 5 min at 4 °C, and a LIVE/DEAD™ Fixable Aqua Dead Cell Stain Kit (Invitrogen) was used to distinguish live cells and dead cells. Cells were incubated with a fluorochrome-labeled antibody cocktail for surface staining. For intracellular staining, cells were fixed by adding fixation buffer (Invitrogen) and permeabilized using permeabilization buffer (Invitrogen) before staining for intracellular targets. Analysis was performed using an Attune NxT flow Cytometer (ThermoFisher, Waltham, MA, USA), and results were analyzed using FlowJo software v10 (BD, Ashland, OR, USA). The principal gating strategy for the respective immune cell subsets is shown in Supplementary Figure S3A. A list of the antibodies used can be found in Supplementary Table S2.

### 2.9. Statistics

Data were analyzed by *t*-test for the comparison of two groups and one-way ANOVA for the comparison of multiple groups using GraphPad Prism 9. All data were presented as the mean ± SD unless otherwise stated. GraphPad Prism 9 was also used for log-rank Mantel–Cox on Kaplan–Meier survival curve. Significance is presented as * *p* < 0.05, ** *p* < 0.01, *** *p* < 0.001, and **** *p* < 0.0001.

## 3. Results

### 3.1. Characterization of Recombinant CD47 and IFN-γ Encoding Dual-Armed MyxV

**Figure 1:**
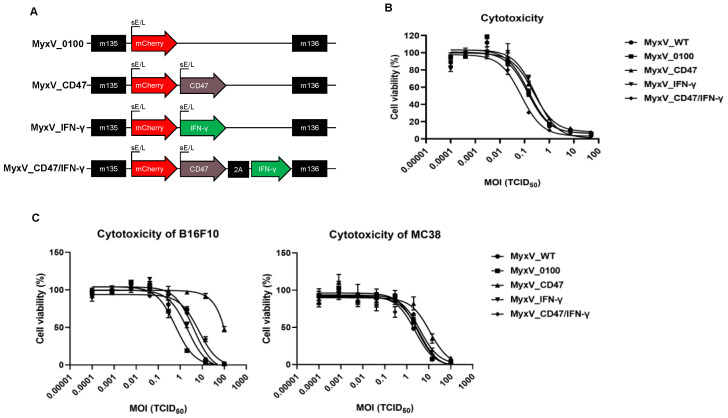
Generation of dual-armed MyxV co-expressing mouse CD47 and IFN-γ. (**A**) Schematic diagram of the armed MyxV genome. The composition of expression cassettes for inserting into MyxV genomes. The indicated M numbers are the open reading frames in the MyxV genome. (**B**) Cytotoxicity was validated on the RK13 cell line after being infected with MyxV_0100 or armed MyxVs at different MOI ranging from 0.0001 to 100 at 5 days post-infection. Cytotoxicity was evaluated by WST assays (Right panel). (**C**) Cytotoxicity evaluated by WST assay was validated on murine cancer cell lines after being infected with MyxV_0100 or armed MyxVs at different MOI ranging from 0.0001 to 100 at 5 days post-infection.

The control MyxV (parental virus—Lausanne strain, obtained from ATCC, hereby referred as MyxV_0100), was constructed by introducing the mCherry fluorescence reporter gene to the intergenic location (between the M135 and M136 genes) under the control of poxvirus early/late synthetic promoter (Figure 1A). We initially assessed the oncolytic activity of the control MyxV_0100 in vitro against four mouse cancer cell lines. MyxV_0100 reliably infected and killed B16F10 and MC38 murine cancer cell lines, while CT26 and 4T1 murine cancer cell lines showed less sensitivity, as shown by cell viability, and virus amplification (Figure S1). The rabbit kidney-derived RK13 cell line was used for virus production and used as control. We next measured the modifications in tumor immunity-related genes caused by MyxV_0100 infection. The four murine cancer cells were infected with MyxV_0100 and expression of the cytokines IFN-γ, GM-CSF, IL-6, IFN-β, and the immune checkpoint-related genes FLT3L, OX40L, PD-L1, and CD47 were found elevated (Figure S2). Collectively, MyxV_0100 showed not only some cytopathic effect on murine cancer cells, but also an increase in the expression of genes that induce immune suppression or regulation of survival for the virus. We got particularly interested to augment further the expression of CD47 and IFN-γ as these two factors could offer improved immunotherapeutic responses to MyxV_0100.

We constructed singly and dual-armed MyxV expressing mouse mCD47 or/and mouse mIFN-γ at the site downstream of the mCherry gene of parental MyxV_0100 under the control of independent promoters (Figure 1A). The four viruses (MyxV_0100, MyxV_CD47, MyxV_ IFN-γ, and MyxV_CD47/IFN-γ) were found to have comparable cytotoxicity in the RK13 cell lines (Figure 1B). In B16F10 and MC38 murine cancer cells, inserting CD47 or/and IFN-γ into MyxV_0100 only slightly interfered with infection and replication (Figure 1C) as compared to the control virus. Interestingly, in B16F10 cancer cells for reasons under investigations, MyxV_IFN-γ showed much-reduced cytotoxicity (Figure 1C left panel).

**Figure 2:**
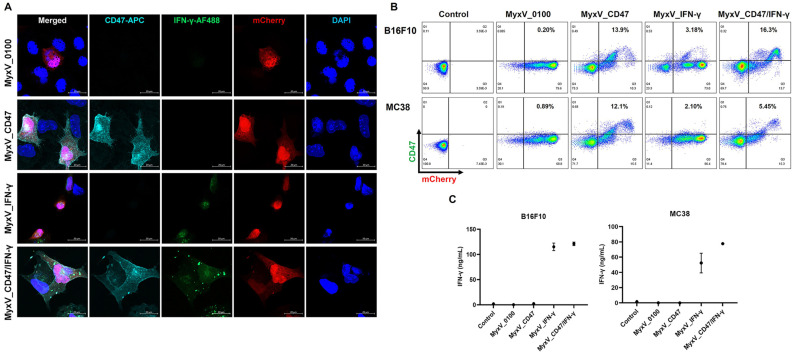
Characterization of dual-armed MyxV co-expressing mouse CD47 and IFN-γ. (**A**) Representative fluorescence microscopic images of B16F10 cells 48 h post-infection with the different MyxVs. CD47 at the cell surface was detected by cyan color, green colors detected IFN-γ in the cytosol, mCherry from MyxV was detected in cytosol and nucleus by red color, and DAPI was used as a counterstain for nuclei and detected by blue color. (**B**) FACS analyses of membrane CD47 after armed MyxVs infection. B16F10 and MC38 cells were infected with 1MOI of MyxVs for 48 h. (**C**) Quantification of conditioned medium for IFN-γ production in armed MyxVs infected B16F10 or MC38 at 48 h post-infection.

To confirm the subcellular distribution of the transgenes, B16F10 cancer cells were infected with the four MyxV and followed by immunofluorescence staining. As expected, CD47 (Pale blue) appeared on the cell surface, and IFN-γ (Green) stained the cytoplasm (Figure 2A). The expression of mCherry (Red) was used as a criterion for virus infection and DAPI nuclear staining used as a control. CD47 membrane expression and secreted IFN-γ expression were also confirmed by FACS analysis and ELISA, respectively (Figure 2B,C). Forty-eight hours post-infection, B16F10 and MC38 cells infected with the appropriate corresponding viruses showed elevated expression of one or both respective transgenes. Together, these data demonstrate that the armed MyxV could infect cancer cells and express similar levels of CD47 and IFN-γ as compared to those in single armed viruses.

### 3.2. Validation of the Anticancer and Immune Stimulation Activity of Dual-Armed MyxV against Murine Melanoma

**Figure 3:**
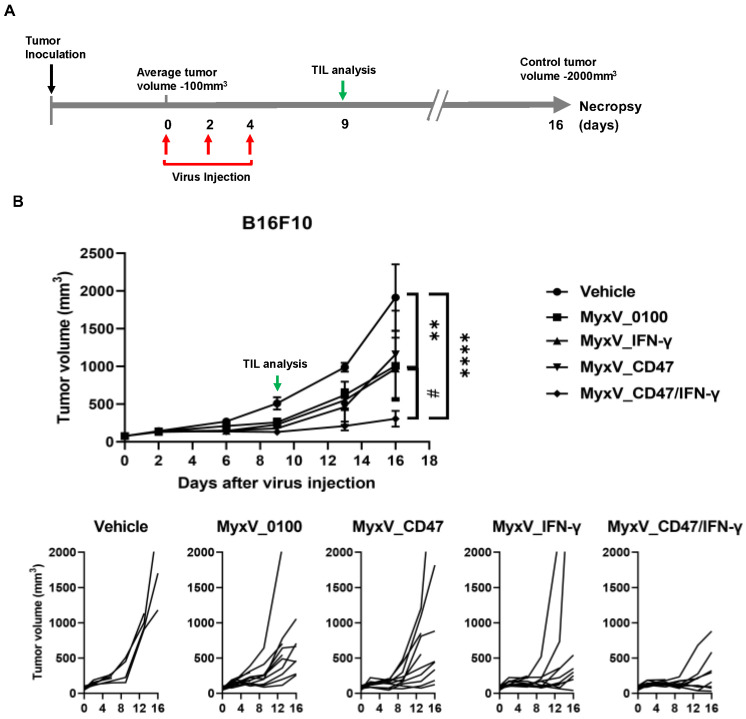
Dual-armed MyxV enhances anti-tumor activities against B16F10 murine melanoma. (**A**) Schematic representation of the experiment with time points for the administration of MyxV. (**B**) Mice were subcutaneously inoculated with B16F10 cells. When tumors reached 100 mm^3^, tumors were directly injected with PBS or MyxVs three times. B16F10 tumor volumes were measured by caliper. Lower graphs show individual mouse tumor growth in each group. Data are presented as means ± SD ****, *p* < 0.0001; **, *p* < 0.01 (Compared with results for the vehicle control group) and #, *p* < 0.05 (Compared with results for MyxV_0100 treated group) by one-way ANOVA.

We next evaluated the therapeutic efficacy of dual-armed MyxV in in vivo experiments using the syngeneic immunocompetent mouse B16F10 model. B16F10 murine melanoma tumors were established subcutaneously at the right flank of C57BL/6 mice, and tumor size was measured twice weekly. Nine days post-virus injection, three mice of each treatment group were sacrificed, and TIL population was compared by FACS analysis (Figure 3A). Intratumoral administration of dual-armed MyxV_CD47/IFN-γ demonstrated significant anticancer efficacy, with average tumor growth inhibitions of 84.1% compared to the vehicle (mock) control group, and 69.6%, 73.6, and 68.4% tumor growth inhibition compared to MyxV_0100, MyxV_CD47, and MyxV_IFN-γ, respectively (Figure 3B).

**Figure 4:**
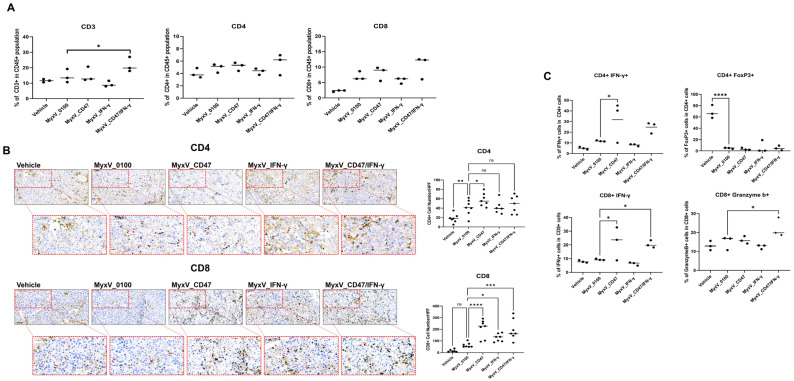
Dual-armed MyxV enhances infiltration and activation of T-cells. (**A**) Two days after the last treatment, tumors were collected and analyzed by FACS to determine the percentage of tumor-infiltrating CD3+, CD4+, and CD8+ T-cells in CD45+ cells. (**B**) Representative immunohistochemical staining for CD4 and CD8 in B16-F10 tumors derived from mice treated with MyxV, stained cells appear in dark brown spots (red arrowhead). Scale bars, 100 μm. Graphs represent tumor-infiltrating CD4 or CD8-positive cells in sections. (**C**) Intratumoral IFN-γ+ T-cells population in B16F10 tumors were measured by FACS. The Granzyme b+ or FoxP3+ population in CD4+ or CD8+ T-cells in B16F10 tumors were measured by FACS. Data are presented as means ± SD ****, *p* < 0.0001; ***, *p* < 0.001; **, *p* < 0.01; *, *p* < 0.05, ns: not significant (Compared with results for the MyxV_0100 treated group) by one-way ANOVA.

Since we did not observe a drastically different viral replicative potentials of the different MyxV in vitro, these results suggest that the increased anticancer efficacy of dual-armed MyxV is not only due to direct cytotoxicity but likely other mechanisms involving activation of anticancer immunity, through changes in immune profile within the tumor microenvironment. Thus, we next evaluated the total population of CD3+, CD4+, and CD8+ T-cells in tumors. As a result of analyzing the immune cells present in the tumor tissues, a significant increase in CD3+ T-cells and CD8+ T-cells was observed in the MyxV_CD47/IFN-γ treatment group. (Figure 4A,B). Additionally, an increased percentage of IFN-γ+ CD8+ and Granzyme b+ CD8+ T-cell population was observed for MyxV_CD47/IFN-γ-treated tumors. Interestingly, the rate of Foxp3+ CD4+ regulatory T-cells (Treg) in CD4+ T-cells was in contrast, decreased in all MyxV-treated groups (Figure 4C). Another interesting result is that MyxV_CD47, contrary to our expectations, evoked more CD4+ T-cells than MyxV_IFN-γ, and even increased the IFN-γ+ CD4+T-cell population in the tumor. These data propose that the overexpression of CD47 on tumor cells may work differently than we expected, and further research is needed on the function of CD47 during tumor virotherapy. In addition, the significant reduction in Treg by MyxV administration suggests that this OV causes substantial changes in the tumor microenvironment, indicating that the development of a combination treatment strategy that could add to this effect could optimize therapeutic efficacy.

### 3.3. Anticancer Efficacy and Prolonged Survival of B16F10 Mouse Melanoma Administered Armed MyxV in Combination with Immune Checkpoint Inhibitor αPD-L1

**Figure 5:**
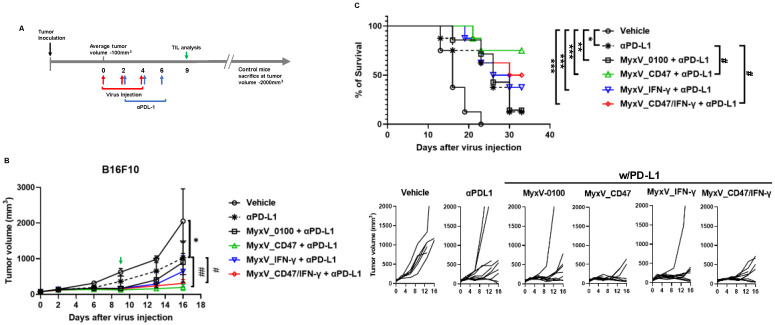
The αPD-L1 combination treatment with armed MyxV enhances anti-tumor activities against B16F10 murine melanoma. (**A**) Schematic representation of the experiment with time points for the administration of MyxV or immune checkpoint inhibitors. (**B**) B16F10 tumor-bearing mice were intratumorally injected with PBS or armed MyxVs, and intraperitoneal injection was performed with αPD-L1. B16F10 tumor volumes were measured by caliper. Lower graphs show individual mouse tumor growth. (**C**) The survival of treated mice was monitored by Kaplan–Meier analysis, and statistical analysis was performed with the Log-rank test. Data are presented as means ± SD ***, *p* < 0.001; **, *p* < 0.01; *, *p* < 0.05 (Compared with results for the vehicle control group) and ##, *p* < 0.01; #, *p* < 0.05 (Compared with results for the αPDL-1treated group) by one-way ANOVA.

We observed that the dual-armed MyxV stimulated the infiltration of lymphocytes within tumors and demonstrated anticancer efficacy. We also found that MyxV infection could augment the expression of PD-L1 on infected mouse tumor cells, thus combining MyxV with immune checkpoint inhibitors could further boost developing anticancer immune responses against systemic tumors. Thus, we examined the therapeutic benefit of combination therapy of the different MyxV with αPD-L1 antibodies against B16F10 syngeneic models. MyxVs were introduced intratumorally, and αPD-L1 was administered via the intraperitoneal route. As in the previous experiment, we selected three mice on the 9th day post-virus injection and performed FACS analyses for TIL population (Figure 5A). Our data showed that a significant reduction in tumor growth rate was confirmed in all treatment groups compared with the vehicle control group, and the αPD-L1 single treatment arm was able to reduce 50.0% of B16F10 melanoma growth compared with vehicle treatme (Figure 5B). As expected, the αPD-L1 and dual-armed MyxV combination therapy group showed significant tumor growth inhibition compared to the αPD-L1 single or MyxV_0100 and αPD-L1 combination treatment (growth inhibition 84.9% vs. vehicle control). Surprisingly, the αPD-L1 with the MyxV_CD47 combination group had the strongest tumor growth inhibitory activity (growth inhibition rate was 90.4% compared to the vehicle control). In addition, MyxV_IFN-γ and αPD-L1 combination treatment did not substantially improved further the tumor growth inhibition compared to the αPDL-1 single or MyxV_0100 and αPD-L1 combination treatment (Figure 5B). The extent of tumor growth inhibition correlated with the overall survival of the treatment groups (Figure 5C).

**Figure 6:**
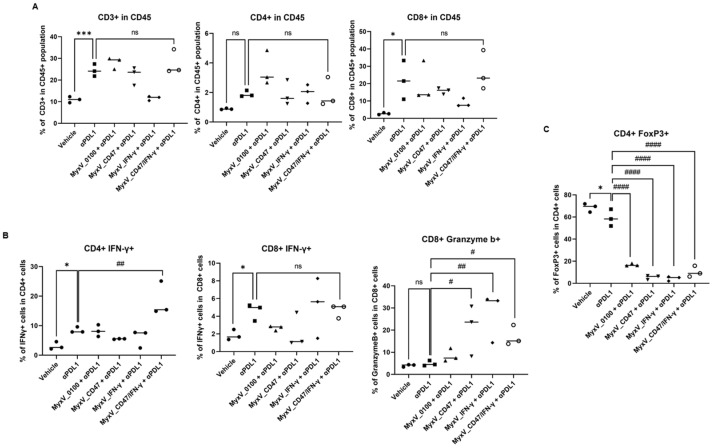
The αPD-L1 combination treatment with armed MyxV alters TIL population and activity. (**A**) Two days after the last treatment, tumors were collected and analyzed by FACS to determine the percentage of tumor-infiltrating CD3+, CD4+, and CD8+ T-cells in CD45+ cells. (**B**) Intratumoral IFN-γ+ or Granzyme b+ population in CD4+ or CD8+ T-cells in B16F10 tumors were measured by FACS. (**C**) The FoxP3+ population in CD4+ T-cells in B16F10 tumors were measured by FACS. Data are presented as means ± SD ***, *p* < 0.001; *, *p* < 0.05, ns: not significant (Compared with results for the vehicle control group) and ####, *p* < 0.0001; ##, *p* < 0.01; #, *p* < 0.05, ns: not significant (Compared with results for the αPDL-1treated group) by one-way ANOVA.

We next analyzed the immune cell infiltration in tumors after combination treatment with αPD-L1 and MyxV. A significant increase in CD3+ and CD8+ T-cells was observed in the αPD-L1 treatment group, and the combination treatment groups compared to the vehicle control group; however, armed MyxV combination treatment did not further increase TIL compared to the αPD-L1 single treatment group (Figure 6A). To further characterize the importance of T-cells in mediating therapeutic responses to combination treatment, tumor-infiltrated T-cells were analyzed. While in the dual-armed MyxV + αPD-L1 treatment group, the ratio of tumor-infiltrated CD4+ T-cells was similar to that of the αPD-L1 single treatment group, the percentage of activated T-cells, IFN-γ+ CD4+ T-cells, but not IFN-γ+ CD8+ T-cells, was significantly increased in tumor infiltrated CD3+ T-cell population in the combination treatment of αPD-L1 and MyxV_CD47/IFN-γ (Figure 6B). In contrast and most interestingly, the proportion of Granzyme b+ CD8+ T-cells or Treg were significantly reduced inside tumors exposed to MyxV (Figure 6B,C). These results indicate that the combination treatment of αPD-L1 and armed MyxV has superior potency in infiltrating immune cells and increases the anticancer activity of infiltrated immune cells. These data propose that the overexpression of virus-encoded CD47 in tumor cells promotes the immune activation mechanism by αPD-L1. Further preclinical experiments will be required to assess the complete efficacy of the dual-armed MyxV.

## 4. Discussion

Oncolytic MyxV engineered to express CD47 and IFN-γ provides a novel approach to control tumors and stimulate infiltration of lymphocytes within the TME. Here we report that 1. Genetically modified MyxV infects and kills cancer cells in vitro. 2. MyxV infection of mouse cancer cells induces the expression of immune modulators. 3. Locally administered MyxV effectively reduced Treg within tumor tissues. 4. Dual-armed MyxV increases infiltration of T-cells, especially CD8+ T-cells, and increases T-cell activity in tumor tissues. Finally, 5. The armed MyxV in combination with αPD-L1 inhibit tumor growth and prolong survival rate of mice with tumors. These observations suggest that genetically engineered myxoma virus warrants further evaluation as an oncolytic agent against cancers.

Preclinical and clinical studies with OVs have reported the immunotherapeutic potential of these anticancer agents. Next-generation viral backbones are being developed to optimize the clinical efficacy of OVs through viral gene modification and introduction of therapeutic transgenes [5,28]. MyxV is the prototypic member of the Leporipoxvirus genus of the Poxviridae family of DNA viruses and has a relatively large genome (161.8 kbp) encoding for 171 viral genes and an efficient recombinant viral system has been developed. An important characteristic of MyxV is that it only causes disease in rabbits without causing any harm to humans or other animals [9]. Therefore, its lack of pathogenicity to hosts other than rabbits, ease of genetic engineering, and susceptibility to a wide range of tumor cells make MyxV an attractive anticancer agent [9,29].

We report here the experimental treatment of murine melanoma B16F10 tumors using a genetically engineered MyxV armed with CD47 and IFN-γ. We initially hypothesized that CD47 overexpressed within infected cancer cells could prolong viral replication by limiting the rapid phagocytosis of virus-infected tumor cells, thereby delaying the production of neutralizing antibodies against the virus and ensuring increased production of other therapeutic transgenes. As an additional transgene, we selected IFN-γ, a potent immune-stimulating anticancer cytokines. While we could not confirm prolong infectivity within tumor tissue due to CD47 expression, the dual-armed MyxV showed significant tumor growth inhibitory effects of the B16F10 syngeneic mouse model compared to parental MyxV and to each single-armed MyxV. Immune stimulation potential confirmed the increased percentage of T-cells population in the tumor, and the population of effector T-cells, IFN-γ+ CD4+, and IFN-γ+ CD8+ T-cells, was also increased. These results suggest that the transgenes CD47 and IFN-γ inserted into dual-armed MyxV can promote anticancer immune responses. Interestingly, coating oncolytic herpes simplex virus with CD47 or on the opposite, targeting CD47 using antibodies encoded within oncolytic viruses has both been successfully developed as approaches to improve the antitumor innate immune responses to cancer [30,31]. Thus, the absence or presence of CD47 can provide beneficial effects on different oncolytic viruses. Whether this is specific to a viral strain would need to be studied in further details. Most interestingly, a significant reduction in Treg inside the tumor was confirmed by intratumorally administered MyxV, an effect within the TME cell population that was not observed by ICI monotherapy. Tregs, one of the T-cell subpopulations, are cells that play an important role in suppressing excessive immune responses and maintaining homeostasis in vivo under normal conditions [32]. Particularly, Tregs in the TME are often pointed out as an important factor inhibiting ICI’s therapeutic effects against solid tumors [33]. Notably, an excessive increase in the number of Tregs in tumors suppresses the immune response to tumor cells and consequently promotes tumor recurrence or metastasis, and significantly reduces the effect of immunotherapeutic agents such as ICI [34,35]. Furthermore, excessive Tregs accumulation in tumors is closely linked to reduced overall survival [36]. While we found here that ICI targets were increased when MyxV infected tumor cells, reducing Tregs within the TME, one of the effects of MyxV, could act to maximize the efficacy of ICI therapy or CAR-T cell therapy in solid tumors as recently observed [15,37,38,39]. A substantiating result in this study was the increase in tumor growth inhibition and overall survival by MyxV-CD47 in combination with ICI, showing an even better tumor control effect than the dual-armed MyxV treatment group. According to the TIL analyses, an increase in the population of CD8+ T-cells was confirmed in both MyxV_CD47 and dual-armed MyxV, and this increase in CD8+ T-cell by MyxV_CD47 treatment was found to be higher than that of MyxV_0100 or MyxV_IFN-γ treated groups. Additional experiments in other cancer models assessing the combination therapy of armed-MyxVs with ICI therapeutics should be considered to determine the scope of this anticancer therapeutic strategy.

This initial study on the dual-armed MyxV could benefit from several optimizations. For one, we need to increase the transgene expression profile of this MyxV platform, and we are currently developing a new parental MyxV viral backbone with improved transgene protein production within infected tumors. Importantly also, determining the exact mechanisms by which MyxV infection influences the tumor microenvironment, from viral replication within cancer cells and from therapeutic transgene expression, and assessing both responses at the organismal level and the single-cell level within the tumors, will be critical to further improve MyxV-based oncolytic virotherapies. Further research and development on armed MyxV should continue for this promising oncolytic viral therapy.

## 5. Conclusions

Our study shows that an oncolytic Myxoma virus (MyxV) encoding CD47 and IFN-γ is effective against cancer cells and enhances anti-tumor immunity in murine cancer models. MyxV_CD47/IFN-γ reduced tumors growth and extended survival as a monotherapy and in combination with αPD-L1. The virus also improved the immune cells profiles with tumors. This suggests that MyxV_CD47/IFN-γ could be further developed as a therapeutic option for solid tumors.

## Data Availability

The data presented in this study are available in the main text, figures, and tables.

## Supplementary Materials

The following supporting information can be downloaded at: https://www.mdpi.com/article/10.3390/cancers15194703/s1, Figure S1: Oncolytic and replication activity of MyxV_0100 in murine cancer cell lines; Figure S2: Expression of cytokines and the immune checkpoint-related genes in MyxV_0100 infected murine cancer cell lines; Figure S3: FACS analyses of tumor-infiltrated T-cell subsets in tumor; Table S1: Primer List; Table S2: Antibody List.
