# Supplementary material for: Dual-Armed Oncolytic Myxoma Virus Encoding IFN-γ and CD47 Promotes Lymphocyte Infiltration and Tumor Suppression of Syngeneic Murine Melanoma
Source: Cancers (Basel). 2023 Sep 24;15(19):4703. doi: 10.3390/cancers15194703 (PMC10571683; doi:10.3390/cancers15194703)
Supplement: Supplementary file 1 [file cancers-15-04703-s001.zip › cancers-2618475-supplementary.pdf]

Supplementary Data

Dual-Armed Oncolytic Myxoma Virus Encoding IFN-γ and CD47 Promotes Lymphocyte Infiltration and Tumor Suppression of Syngeneic Murine Melanoma

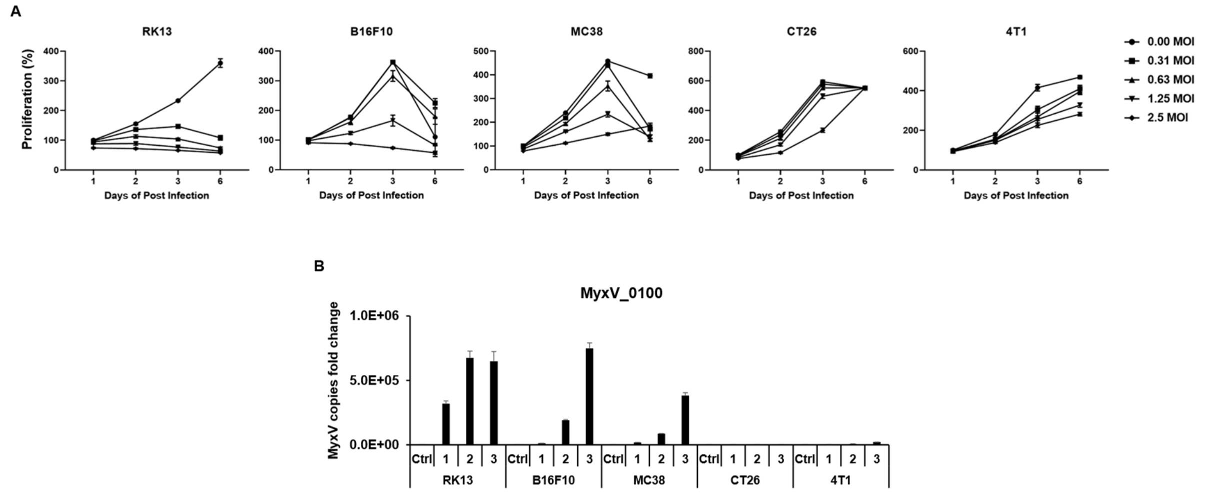

Figure S1. Oncolytic and replication activity of MyxV_0100 in murine cancer cell lines

A. Graphs show the relative proliferation rates of four different murine cancer cell lines and a rabbit-derived cell line RK13 when infected with different MOI of MyxV_0100 for 1-, 2-, 3-, and 6- days post-infection, respectively. **B**. RK13 and murine cancer cell lines B16F10, MC38, CT26, and 4T1 were used for virus amplification. The cells were infected with 0.2MOI of MyxV_0100, and PCR analysis was conducted at 1, 2, and 3 days of post-infection. Virus amplification was compared with the uninfected control. β-actin was used as an internal control.

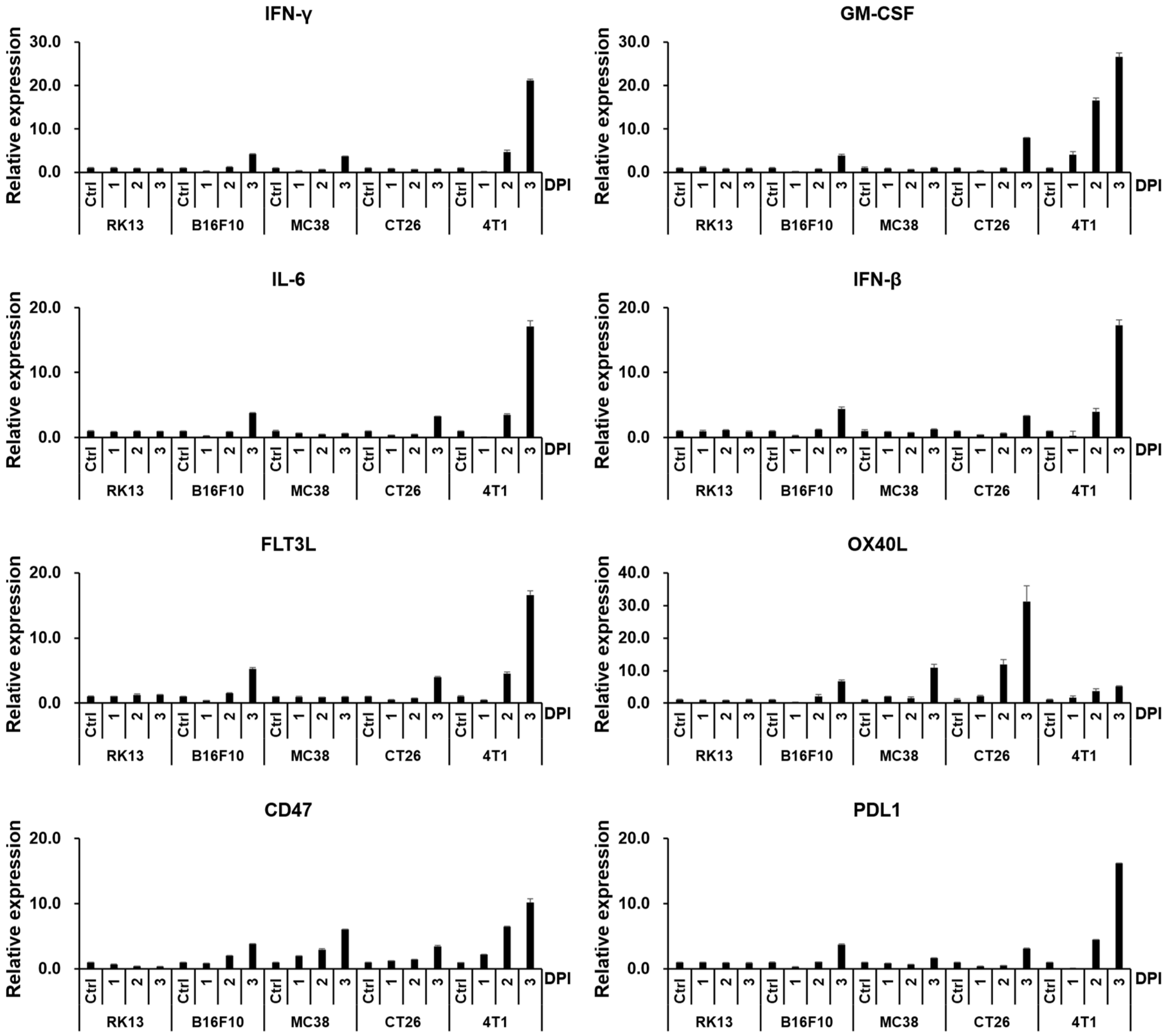

Figure S2. Expression of cytokines and the immune checkpoint-related genes in MyxV_0100 infected murine cancer cell lines

RK13 and murine cancer cell lines B16F10, MC38, CT26, and 4T1 were used for qPCR analysis. The cells were infected with 0.2MOI of MyxV_0100, and qPCR analysis was conducted at 1-, 2-, and 3-days post-infection. The expression of cytokines or cell receptors was compared with the uninfected control. β-actin was used as an internal control.

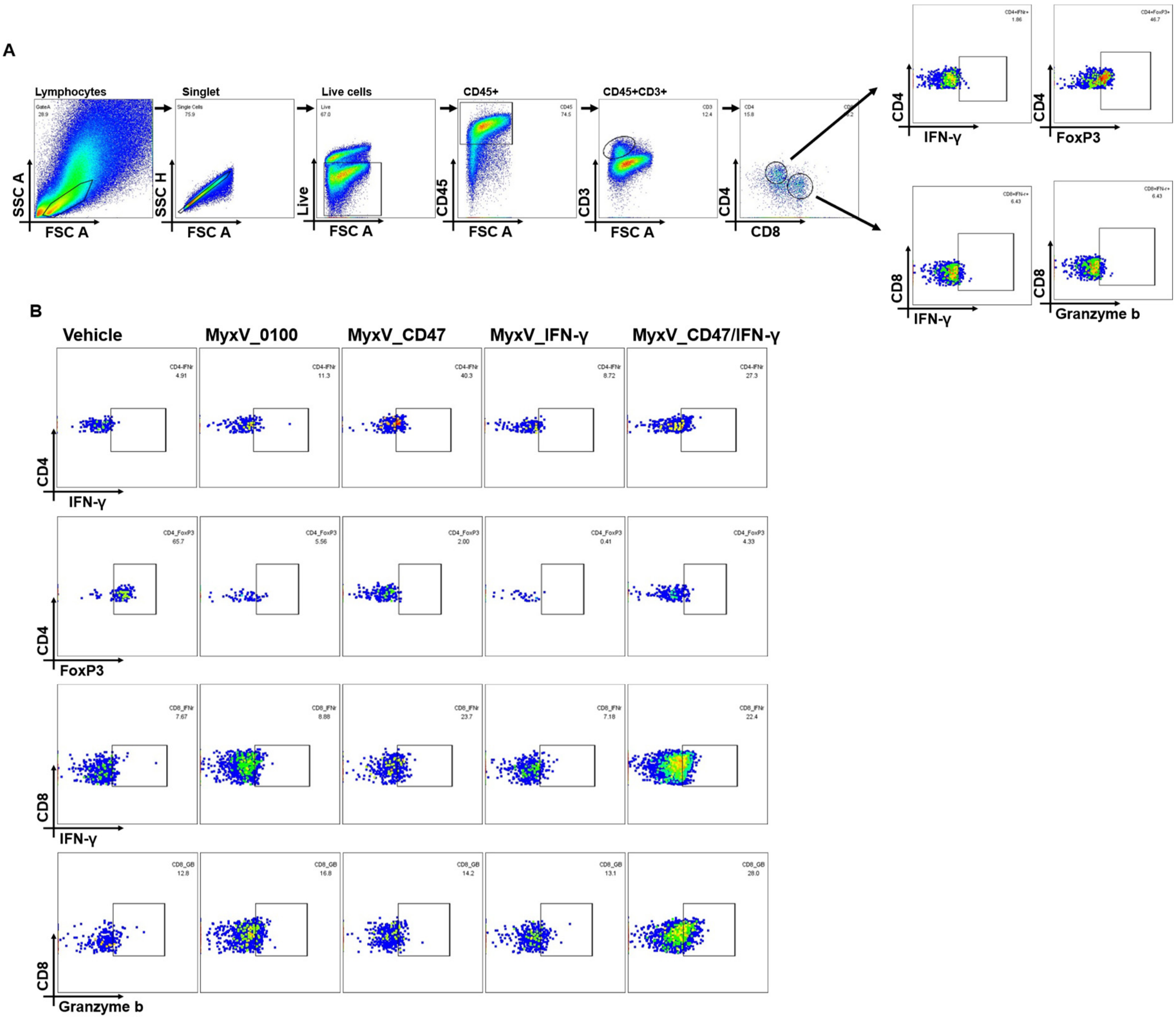

Figure S3. FACS analyses of tumor-infiltrated T-cell subsets in tumor.

A. Gating strategy for phenotypic analysis of T-cell subsets in the tumor. B. Representative FACS plot of T-cell subsets (CD4+ IFN-γ+, CD4+ Foxp3+, CD8+ IFN-γ+, and CD8+ Granzyme b +).

Table S1. Primer List

Gene	5′ → 3′	Sequence	Size (bp)	Gene ID
**Myxoma virus**	**Forward**	**GTTGGTAATTTCATTTCGTACCA**	**119**	**NC_001132.2**
	**Reverse**	**GGTAAACAATTAATCCGTTGTAA**		
**IFN-γ**	**Forward**	**AGCCTCAGGAAGCGGAAAAG**	**354**	**NM_008337.4**
	**Reverse**	**TGAGTTCAGTCAGCCGCTTG**		
**GM-CSF**	**Forward**	**GGCTGCAGAATTTACTTTTCCTGGG**	**366**	**NM_009969.4**
	**Reverse**	**GGCTGTCTATGAAATCCGCATAGG**		
**IL-6**	**Forward**	**GAGGATACCACTCCCAACAGACC**	**141**	**NM_031168.2**
	**Reverse**	**AAGTGCATCATCGTTGTTCATACA**		
**IFN-β**	**Forward**	**CAACAGGTGGATCCTCCACG**	**446**	**NM_010510.2**
	**Reverse**	**GTTTAAGGTACCTTTGCACCCTCC**		
**FLT3L**	**Forward**	**CAGCCTGGAGCCCAAATTCC**	**599**	**NM_001402831.1**
	**Reverse**	**CAGCAGCACCAGTGTGAGAGG**		
**OX40L**	**Forward**	**GGAAGGGGTTCAACCCCTGG**	**543**	**U12763.1**
	**Reverse**	**GCACAGTATCCAGGCGTTAGC**		
**CD47**	**Forward**	**CCATATTGATCACTCAAGTGC**	**129**	**AB012693.1**
	**Reverse**	**GTTCTGCTAGAGCTATGATC**		
**PD-L1**	**Forward**	**GCTGGCATTATATTCACAGC**	**378**	**GQ904196.1**
	**Reverse**	**GACTTTCAGCGTGATTCG**		
**β-Actin**	**Forward**	**TGGAATCCTGTGGCATCCATGAAAC**	**349**	**NM_007393.5**
	**Reverse**	**TAAAACGCAGCTCAGTAACAGTCCG**		

Table S2. Antibody List

Antibody Name	Clone	Company	Cat No.	Usage
Anti-CD47 APC	miap301	eBioscience	17-0471-82	IF
Anti-CD47 FITC	miap301	eBioscience	11-0471-82	FACS
Anti-IFN-γ PE	XMG1.2	eBioscience	12-7311-82	IF
Anti-IFN-γ APC	XMG1.2	eBioscience	17-7311-82	FACS
Anti-CD45 Super Bright™ 780	30-F11	eBioscience	78-0451-82	FACS
Anti-CD3e APC-eFluor™ 780	145-2C11	eBioscience	47-0031-82	FACS
Anti-CD4 FITC	GK1.5	eBioscience	11-0041-82	FACS, IF
Anti-CD8a Super Bright™ 645	53-6.7	eBioscience	64-0081-82	FACS, IF
Anti-FoXP3 eFluor™ 450	FJK-16s	eBioscience	48-5773-82	FACS
Anti-Granzyme B PerCP-eFluor™ 710	NGZB	eBioscience	46-8898-82	FACS
Anti-CD8 alpha antibody	EPR21769	abcam	ab217344	IHC
Anti-CD4 antibody	EPR19514	abcam	ab183685	IHC
Anti-CD11c antibody Alexa Fluor® 594	N418	Biolegend	117346	IF
